# Biofunctional Constituents from *Liriodendron tulipifera* with Antioxidants and Anti-Melanogenic Properties

**DOI:** 10.3390/ijms14011698

**Published:** 2013-01-15

**Authors:** Wei-Jen Li, Yi-Chieh Lin, Pei-Fang Wu, Zhi-Hong Wen, Po-Len Liu, Chung-Yi Chen, Hui-Min Wang

**Affiliations:** 1 Bachelor Degree Program of Health Beauty, School of Medical and Health Sciences, Fooyin University, Ta-Liao District, Kaohsiung 83102, Taiwan; E-Mail: mt082@mail.fy.edu.tw; 2 Department of Fragrance and Cosmetic Science, Kaohsiung Medical University, Kaohsiung 807, Taiwan; E-Mails: yejio5421@hotmail.com (Y.-C.L.); s1988618@yahoo.com.tw (P.-F.W.); 3 Department of Marine Biotechnology and Resources, Asia-Pacific Ocean Research Center, National Sun Yat-sen University, Kaohsiung 80424, Taiwan; E-Mail: wzh@mail.nsysu.edu.tw; 4 Department of Respiratory Therapy, College of Medicine, Kaohsiung Medical University, 100, Shih-Chuan 1st Road, San-Ming District, Kaohsiung 807, Taiwan; E-Mail: kisa@kmu.edu.tw

**Keywords:** *Liriodendron tulipifera*, antioxidant, tyrosinase inhibitor, B16F10

## Abstract

From the stems of *Liriodendron tulipifera*, seventeen known compounds have been extracted, isolated and purified. By using spectroscopic analysis, the structures of these pure constituents were determined as three lignans, four steroids and ten benzenoids. Identified compounds were screened for antioxidant abilities using: 1,1-diphenyl-2-picrylhydrazul (DPPH) and 2,2′-azino-bis(3-ethylbenzothiazoline-6-sulfonic acid) (ABTS) scavenging free radical activity assays; metal chelating power test; and ferric reducing/antioxidant power (FRAP) examination. The result revealed that seventeen compounds had potential anti-oxidative capabilities. In addition, the anti-tyrosinase effect was determined by calculating the hydroxylation of L-tyrosine to L-dopa and the oxidization of L-dopa to dopaquinone, according to *in vitro* mushroom tyrosinase evaluation platform. Furthermore, based on assays on B16F10 cell line, our data suggest that five compounds isolated from *L. tulipifera* would be able to inhibit tyrosinase activity and reduce the melanin content in animal cells. Therefore, some of the examined compounds could be potentially used in the cosmetic skin whitening business, therapeutic applications or the food industry.

## 1. Introduction

*Liriodendron tulipifera* is a fast growing hardwood native plant in the United States, usually used for pulp and wood in furniture and paper-making proposes [1,2]. In earlier investigations of *L. tulipifera* chemical constituents, some sesquiterpenes and aporphine alkaloids were discussed [3]. Previous studies found that some compounds isolated from *L. tulipifera* showed anti-migration abilities and inhibited the proliferation of cancer cells. DPPH, chelating and reducing power assay also revealed its antioxidant effects. Thus, we assume the compounds isolated from *L. tulipifera* have promising multi-biofunctions. For this reason, *L. tulipifera* was chosen for further phytochemical investigations, and these compounds were isolated from the stems. The MeOH extracts of this plant were subjected to solvent partitioning and chromatographic separation to generate seventeen pure substances. The chemical constituents in the plants of *L. tulipifera* were separated by using column chromatography, including three lignans: (−)-eudesmin (**1**) [4], (+)-syringaresinol (**2**) and (+)-yangambin (**3**) [5]; four steroids: β-sitosterol (**4**), stigmasterol (**5**) [6], β-sitostenone (**6**) and stigmastenone (**7**) [7]; and ten benzenoids: methyl 4-hydroxy-2-methylbenzoate (**8**) [8], methyl β-orcinol carboxylate (**9**), methyl haematommate (**10**) [9], coniferyl aldehyde (**11**) [10], vanillin (**12**), vanillic acid (**13**), methyl vanillate (**14**), *p*-hydroxybenzoic acid (**15**), syringic acid (**16**) and 2,6-dimethoxy-*p*-quinone (**17**) [11].

Free radicals and reactive oxygen species (ROS) were produced from the formation of hydrogen peroxide or superoxide anions. They will attack some important biological molecules, such as DNA, protein or lipids and enhance oxidative stress in human beings [12,13]. Evidence indicates that many degenerative disease conditions, such as cancer, gastric ulcers, Alzheimer’s, arthritis and ischemic reperfusion are caused by free radicals or active oxygen species [14,15]. In recent years, ingesting fresh fruits, vegetables or teas to acquire natural antioxidants have been purported to benefit human health. Dietary strategies are considered important to prevent diseases [16,17]. Natural antioxidant constituents, capable of lowering free radical mediated degradations of cells and tissues in human organism, are very substantial in cosmetics or food businesses [18,19]. Thus, much attention has shifted to effective components present in a natural plant-based diet. According to our previous studies, we found a great amount of various phytochemicals possess antioxidative, skin whitening or chemotherapeutic activities [6,12–15,18–20].

The color of skin, hair and eyes of mammals are determined by synthesis and distribution of melanin. There are two major types of melanin occuring in mammals: black/brown eumelanins and yellow/red pheomelanins. In melanogenesis, L-tyrosine is hydroxylated to dihydroxyphenylalanine (L-dopa), and L-dopa is oxidized to dopaquinone followed by further conversion to melanin production. Tyrosinase is the first and rate limiting enzyme in melanogenesis, hence, inhibition of tyrosinase is one of the major strategies to treat hyperpigmentation [21]. In clinical usage, whitening agents are used for treating dermatological disorders related to melanin hyper-accumulation, such as melasma, freckles, lentigo and pigmented acne scars. Besides, they are essential in cosmetics for depigmentation. Thus, the depigmenting agents are very important in both cosmetics and medicinal industries [22].

**Figure 1:**
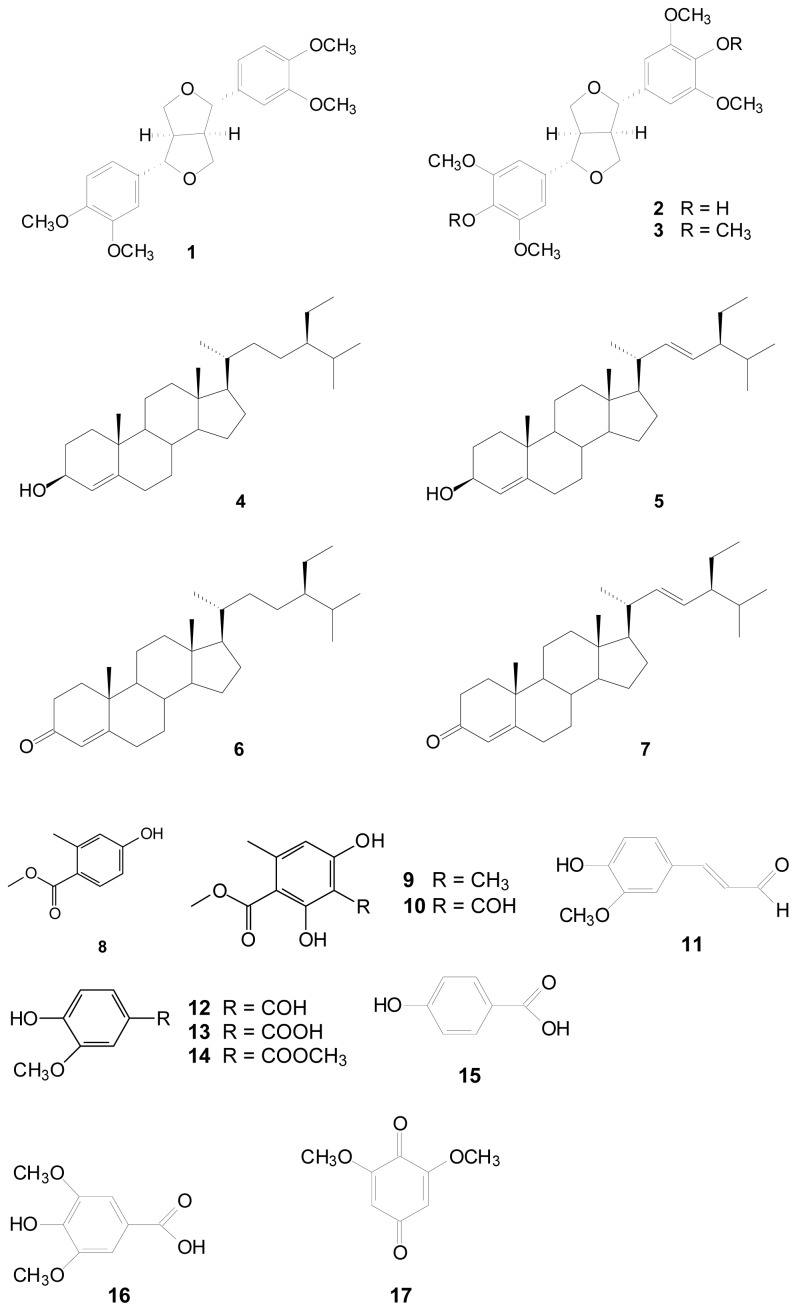
The chemical structures of compounds **1**–**17** from the stems of *L. tulipifera*.

The aim of this current work was to evaluate the antioxidant activities and anti-melanogenic potential of our newly isolated compounds from *L. tulipifera*. The chemical structures of the **17** compounds isolated from *L. tulipifera* were shown in Figure 1. Different non-enzymatic anti-oxidative testing methodologies were used to determine the antioxidant activities of **1**–**17**, such as 1,1-diphenyl-2-picryl-hydrazyl (DPPH) and 2,2′-azinobis(3-ethylbenzothiazoline-6-sulfonic acid) (ABTS) free radical scavengings, metal chelating powers and ferric reducing/antioxidant power (FRAP) activities. The inhibition effects of *L. tulipifera* components were studied both *in vitro* on mushroom tyrosinase and *in vivo* on B16F10 cells to evaluate their skin whitening potential for cosmetic functions or in pharmaceutical applications.

## 2. Results and Discussion

### 2.1. Antioxidant Activities of Compounds **1** to **17** from *Liriodendron tulipifera*

#### 2.1.1. DPPH Free Radical Scavenging Activity Assay

DPPH free radical scavenge is an acknowledged, easy and cheap testing system by which antioxidants act to inhibit oxidation products. Hence, it is used widely as one of the norms for antioxidant activity, and antioxidants were able to change the stable radical DPPH purple solution color to light yellow diphenyl-picrylhydrazine. To investigate the antioxidant activities of *L. tulipifera* compounds **1**–**17**, a dosage of 100 μM was used to determine their scavenging properties. As shown in Table 1, only compound **2** exhibited a moderate radical scavenging action (38.5%), while vitamin C at the same condition (100 μM) resulted in 88.6% activity.

#### 2.1.2. ABTS^+^ Cation Radical Scavenging Assay

The anti-oxidative activities of *L. tulipifera* compounds were determined by the de-colorization of the ABTS^+^, gained through measuring absorbance at 734 nm. The reducing power was determined by the radical cation as the percentage inhibition. Table 1 illustrated that the middle-high scavenging effects of compounds **1** (64.1%), **2** (84.8%), **6** (52.4%), **9** (79.8%) and **10** (72.5%) on the suppression of the absorbance of the ABTS^+^ radical cations. In addition, compounds **3** (28.1%), **12** (19.4%), **13** (22.7%), **16** (49.4%) and **17** (31.5%) exhibited minor inhibition activities within this assay, and residual compounds did not possess ABTS^+^ radical scavenging properties. Vitamin C showed 76.4% on ABTS^+^ cation radical scavenging assay at 100 μM.

#### 2.1.3. Ferrous Ions Chelating Capacity

The ferrous ion chelating activities of *L. tulipifera* compounds were reported in Table 1. EDTA (100 μM) was used as a positive control. Ferrozine and Fe^2+^ can quantitatively form complexes. In the presence of chelating agents, the reagent complex formation is disrupted, resulting in a reducing in the dark red color of the complex. Compounds **3**, **8**, **10** and **17** at the dosage of 100 μM presented minor levels on Fe^2+^ scavenging effects of 13.5%, 17.4%, 19.8% and 25.5%, respectively. EDTA possessed 86.9% ion chelating capacity at 100 μM.

#### 2.1.4. FRAP Power

Ferric reducing antioxidant power assay is a reliable and common test to measure the reducing potential of an antioxidant reacting with a ferric 2,4,6-tripyridyl-*S*-triazine (Fe(III)-TPTZ) complex, which produces a dark blue colored ferrous Fe(II)-TPTZ complex from an adopted reductant. Depending on the reducing power of these antioxidants, the color of the testing solutions changed from different shades of green and blue. This complex has a noticeable dark color that can be detected at 700 nm when there is a higher absorbance, which means a higher ferric reducing power. In Table 1, compounds **2** and **9** exhibited middle-higher ferric reducing power, and **1**, **3**, **6**, **8**, **10**–**13**, **16** and **17** presented minor absorbance. BHA showed 0.98 on FRAP power assay at 100 μM.

As we know, the antioxidant strength of a compound depends on the related replaced hydroxyl or phenolic types. Through the structure activity relationship (SAR) studies, we found that compounds **2** and **9** had one or more of the hydroxyl groups, and therefore, **2** and **9** had good antioxidative activities. Compound **2** had the biphenyl symmetry structure, and the ortho position had two methoxyl groups on the hydroxyl positions. Compound **9** belonged to the phenol type containing two hydroxyl groups, and between the hydroxyl groups was a methyl group. This study demonstrated that di-hydroxyl groups have stronger antioxidant capacity than the single hydroxyl group. The hydroxy-ortho-methoxyl group (compound **2**) and hydroxy-ortho-alkyl group (compound **9**) structures increased the antioxidant activities.

### 2.2. *In vitro* Mushroom Tyrosinase Inhibition

In melanin synthesis, tyrosinase catalysis has two distinct reactions: the hydroxylation of L-tyrosine to L-dopa and the oxidation of L-dopa to dopaquinone. Dopaquinone after inherent conversion becomes dopachrome, and dopachrome tautomerase (tyrosinase-related protein-2, DCT/TRP-2) catalyzes the conversion of dopachrome to 5,6-dihydroxyindole-2-carboxylic acid (DHICA). By DHICA oxidase (TRP-1), DHICA is converted to indole-quinone-carboxylic acid. The tyrosinase-related proteins, TRP-1, and TRP-2 catalyze distal melanin synthesis steps, which control the type of melanin produced [21,22]. After we added the L-tyrosine as the substrate, the serial reaction was the hydroxylation of L-tyrosine to L-dopa and the oxidation of L-dopa to dopaquinone. We tested the dopaquinone at 490 nm, and the final concentration of DMSO was at 0.5%.

We measured 17 compounds from *L. tulipifera* on *in vitro* mushroom tyrosinase inhibition platform to find effective and new substances for melanogenesis inhibitors, the prevention of hyper-pigmentation and skin whitening. Fortunately, we discovered four compounds **8**, **13**, **15** and **16** at 100 μM that showed minor-middle levels on reducing mushroom tyrosinase activity of 23.2%, 22.8%, 15.4% and 10.6%, respectively. Kojic acid inhibited 89.2% of mushroom tyrosinase. (Table 2) indicates that these compounds have potential utilization to inhibit melanin synthesis.

### 2.3. Cytotoxicity of *L. tulipifera* Compounds in B16F10 Cells

**Figure 2:**
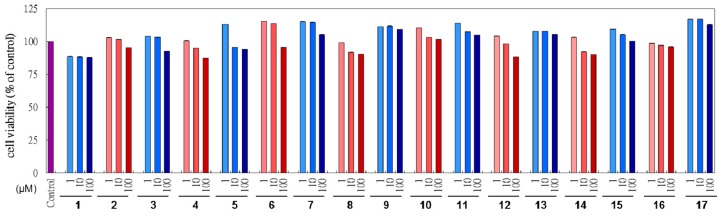
The B16F10 cell viability with various doses (1, 10, 100 μM) of *L. tulipifera* compounds were examined using MTT assay. The control group had no additional agents. *p <* 0.05.

3-(4,5-Dimetylthiazol-2-yl)-2,5-diphenyl tetrazolium bromide (MTT) assay was investigated to study the cytotoxicities of *L. tulipifera* compounds in B16F10 cells. The samples were treated with various concentrations, from 1 μM to 100 μM, and the vehicle control group had no testing agents with 0.5% DMSO. As shown in Figure 2. The compounds isolated from *L. tulipifera* showed no apparent cytotoxicity. Therefore, they have the potential to be safe and effective agents.

### 2.4. Cell Based Examination on *L. tulipifera* Compounds

**Figure 3:**
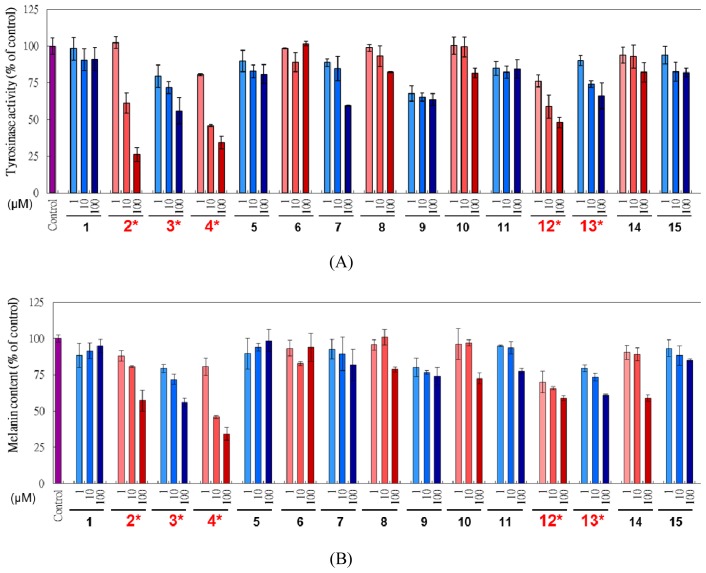
Tyrosinase activities (**A**) and melanin contents (**B**) of B16F10 cells treated with various concentrations (1, 10, 100 μM) of the compounds isolated from *L. tulipifera* for 48 h culture. The control group had no additional agents. *p* < 0.05.

Melanin is a vitally important factor in determining the skin color of human. The melanogenesis pathway consists of the enzymatic L-tyrosine hydroxylation and the oxidation of L-dopa to its corresponding dopaquinone. After the two tyrosinase-catalyzed steps, additional multiple biosynthesis steps followed and yield melanin [17]. *L. tulipifera* compounds were investigated for mouse B16F10 cellular tyrosinase-inhibiting abilities (Figure 3A) and melanin content (Figure 3B) decreasing powers. By increasing the concentration of testing compounds from 1 to 100 μM, most tyrosinase activities and melanin contents decreased. We discovered that the melanin contents matched with the tyrosinase activities in the same dose-dependent tendencies, which meant the cellular melanin reductions might be due to the inhibition of tyrosinase activities. Combining the results of tyrosinase activities and melanin contents, compounds **2**, **3**, **4**, **12** and **13** had the most apparent inhibition abilities. According to the results, those compounds might have a potential in skin lightening properties. In mushroom tyrosinase, we found that inhibitory effectiveness of 17 compounds was not coincident with tyrosinase activities and melanin contents in B16F10 assay. There are many reported literatures using mushroom tyrosinase as a platform model to show the tyrosinase activity. However, mushroom tyrosinases have significant differences from mammalian tyrosinases in catalysis mechanisms [22]. This phenomenon explained that diverse species might cause various effects of enzyme reactions.

## 3. Experimental Section

### 3.1. General Procedure

UV spectra were obtained in MeCN. IR spectra were measured on a Hitachi 260-30 spectrophotometer. ^1^H NMR (400/500 MHz), ^13^C NMR (100 MHz), HSQC, HMBC, COSY and NOESY spectra were obtained on a Varian (Unity Plus) NMR spectrometer. For each sample, 128 scans were recorded with the following parameter: 0.187 Hz/point; spectra width, 14,400 Hz; pulse width, 4.0 μs; relaxation delay, 2 s. Low-resolution ESI-MS spectra were obtained on an API 3000 (Applied Biosystems, Foster City, CA, USA) and high-resolution ESI-MS spectra on a Bruker Daltonics APEX II 30e spectrometer. Silica gel 60 (Merck, 70~230 mesh, 230~400 mesh, Darmstad, Germany) was used for column chromatography. Precoated Silica gel plates (Merck, Kieselgel 60 F-254, Darmstad, Germany), 0.20 mm and 0.50 mm, were used for analytical TLC and preparative TLC, respectively, and visualized with 10% H_2_SO_4_.

### 3.2. Plant Material

The specimen of *L. tulipifera* was collected from Chiayi County, Taiwan in December, 2007. A voucher specimen was characterized by Dr. Jin-Cherng Huang of the Department of Forest Products Science and Furniture Engineering, National Chiayi University, Chiayi, Taiwan and deposited in the School of Medical and Health Sciences, Fooyin University, Kaohsiung County, Taiwan.

### 3.3. Extraction Isolation and Identification

The air-dried stems of *L. tulipifera* (9.0 kg) were extracted with MeOH at room temperature, and the MeOH extract (187.5 g) was obtained upon concentration under reduced pressure. The MeOH extract was chromatographed over silica gel using CH_2_Cl_2_/MeOH as eluent to produce seven fractions. Fraction 1 was subjected to Si gel chromatography by eluting with *n*-hexane/acetone to obtain methyl 4-hydroxy-2-methylbenzoate (**8**) (19 mg), methyl β-orcinol carboxylate (**9**) (37 mg), methyl haematommate (**10**) (20 mg), vanillin (**12**) (19 mg), vanillic acid (**13**) (15 mg) and a mixture of β-sitostenone (**6**) and stigmastenone (**7**) (0.94 g). Part of fraction 2 was subjected to Si gel chromatography by eluting with *n*-hexane/acetone to yield (+)-syringaresinol (**2**) (45 mg), (+)-yangambin (**3**) (36 mg), *p*-hydroxybenzoic acid (**15**) (6mg), syringic acid (**16**) (9 mg) and a mixture of β-sitosterol (**4**), and stigmasterol (**5**) (1.72 g). Part of fraction 3 was subjected to Si gel chromatography by eluting with *n*-hexane/acetone to obtain eudesmin (**1**) (27 mg). Part of fraction 4 was subjected to Si gel chromatography by eluting with *n*-hexane/acetone to obtain coniferyl aldehyde (**11**) (14 mg), methyl vanillate (**14**) (9 mg) and 2,6-dimethoxy-*p*-quinone (**17**) (3 mg). These compounds were obtained and characterized by the comparison of their physical and spectral data (UV, IR, NMR and MS) with values obtained in the literature.

(−)-Eudesmin (**1**). White powder (CH_2_Cl_2_); UV λ_max_: 210, 233, 280 nm; IR ν_max_: 1600, 1515 cm^−1; 1^H NMR (500 MHz, CDCl_3_): δ 3.77 (2H, m, H-1 and H-5), 3.90 (2H, m, H-4_axia._ and H-8_axia._), 3.98 (12H, s, OCH_3_), 4.06 (2H, dd, *J* = 8.0, 2.0 Hz, H-4_equ._ and H-8_equ._), 4.79 (2H, d, *J* = 6.5 Hz, H-2 and H-6), 6.69 (4H, s, H-2′, H-2″, H-6′ and H-6″); ESI-MS *m*/*z*: 386 [M]^+^.

(+)-Syringaresinol (**2**). White powder (CH_2_Cl_2_); UV λ_max_ 220, 238, 280 nm; IR ν_max_ 3400, 1610, 1505 cm^−1; 1^H NMR (400 MHz, CDCl_3_): δ 3.09 (2H, m, H-1 and H-5), 3.88 (12H, s, OCH_3_), 4.27 (2H, m, H-4_equ._ and H-8_equ._), 3.91 (2H, d, *J* = 4.0 Hz, H-4_axia._ and H-8_axia._), 4.72 (2H, d, *J* = 4.4 Hz, H-2 and H-6), 5.58 (2H, s, OH), 6.57 (4H, s, H-2′, H-2″, H-6′ and H-6″); ESI-MS *m*/*z*: 418 [M]^+^.

(+)-Yangambin (**3**). White powder (CH_2_Cl_2_); UV λ_max_ 210, 235, 275 nm; IR ν_max_ 1610, 1505 cm^−1; 1^H NMR (500 MHz, CDCl_3_): δ 3.11 (2H, m, H-1 and H-5), 3.84 (6H, s, OCH_3_), 3.88 (12H, s, OCH_3_), 3.95 (2H, dd, *J* = 9.0, 3.5 Hz, H-4_axia._ and H-8_axia._), 4.31 (2H, m, H-4_equ._ and H-8_equ._), 4.75 (2H, d, *J* = 4.0 Hz, H-2 and H-6), 6.58 (4H, s, H-2′, H-2″, H-6′ and H-6″); ESI-MS *m*/*z*: 446 [M]^+^.

β-Sitosterol (**4**) & Stigmasterol (**5**). White needles (CH_2_Cl_2_); UV λ_max_: 205 nm; IR ν_max_: 3420, 2910, 1625, 1450 cm^−1; 1^H NMR (400 MHz, CDCl_3_): δ 0.67 (3H, s, H-18), 0.79 (3H, d, *J* = 6.8 Hz, H-26), 0.82 (3H, d, *J* = 6.8 Hz, H-27), 0.86 (3H, t, *J* = 7.0 Hz, H-29), 0.92 (3H, d, *J* = 6.5 Hz, H-21), 1.02 (3H, s, H-19), 3.59 (1H, m, H-3), 5.02 (1H, dd, *J* = 15.2, 8.8 Hz, H-22), 5.16 (1H, dd, *J* = 15.2, 8.8 Hz, H-23); ESI-MS *m*/*z*: 414 [M]^+^, 412 [M]^+^.

β-Sitostenone (**6**) & Stigmastenone (**7**). White needles (CH_2_Cl_2_); UV λ_max_: 243 nm; IR ν_max_: 1680, 1618, 1460, 1375 cm^−1; 1^H NMR (500 MHz, CDCl_3_): δ 0.69 (3H, *s*, H-18), 0.82 (3H, *d*, H-26), 0.84 (3H, d, *J* = 6.8 Hz, H-27), 0.87 (3H, t, *J* = 7.0 Hz, H-29), 0.95 (3H, d, *J* = 5.6 Hz, H-21), 1.03 (3H, s, H-19), 3.62 (1H, m, H-3), 5.14 (1H, dd, *J* = 15.0, 9.0 Hz, H-22), 5.01 (1H, dd, J = 15.0, 9.0 Hz, H-23), 5.73 (1H, d, J = 1.4 Hz, H-3); ESI-MS m/z: 413 [M]^+^, 410 [M]^+^.

Methyl 4-hydroxy-2-methylbenzoate (**8**). Colorless needles (CH_2_Cl_2_); UV λ_max_: 225, 265, 305 nm; IR ν_max_: 3400, 1670, 1590, 1515 cm^−1^; ESI-MS *m/z* 189 [M+Na]^+^; HR-ESI-MS *m*/*z* 189.0525 [M+Na]^+^ (calcd for C_9_H_10_O_3_Na, 189.0528); ^1^H NMR (500 MHz, CDCl_3_): δ 2.57 (3H, s, C_2_–CH_3_), 3.96 (3H, s, C_4_-OCH_3_), 6.06 (1H, s, OH), 6.95 (1H, d, *J* = 8.5 Hz, H-6), 7.55 (1H, d, *J* = 1.8 Hz, H-3), 7.55 (1H, dd, *J* = 8.5, 1.8 Hz, H-5); ^13^C NMR (125 MHz, CDCl_3_): 26.2 (*C*H_3_), 56.1 (O*C*H_3_), 109.7 (C-5, CH), 113.7 (C-6, CH), 124.0 (C-3, CH), 130.2 (C-1, C), 146.6 (C-2, C), 150.3 (C-4, C), 196.8 (C=O).

Methyl β-orcinol carboxylate (**9**). Colorless needles (CH_2_Cl_2_); UV λ_max_: 220, 236, 317 nm; IR ν_max_: 3400, 1690, 1590, 1515 cm^−1; 1^H NMR (400 MHz, CDCl_3_): δ 2.10 (3H, s, C_3_-CH_3_), 2.45 (3H, s, C_6_-CH_3_), 3.92 (3H, s, COOCH_3_), 5.30 (1H, s, C_4_-OH), 6.21 (1H, s, H-5), 12.04 (1H, s, C_2_-OH); ESI-MS *m*/*z*: 196 [M]^+^.

Methyl haematommate (**10**). Colorless needles (CH_2_Cl_2_); UV λ_max_: 222, 235, 320 nm; IR ν_max_: 3400, 1712, 1670, 1585, 1512 cm^−1; 1^H NMR (400 MHz, CDCl_3_): δ 2.53 (3H, s, C_6_–CH_3_), 3.96 (3H, s, COOCH_3_), 6.29 (1H, s, H-5), 10.34 (1H, s, CHO), 12.41 (1H, s, C_2_–OH), 12.88 (1H, s, C_4_–OH); ESI-MS *m*/*z*: 210 [M]^+^.

Coniferyl aldehyde (**11**). Colorless oil; UV λ_max_: 337, 305, 238, 222 nm; IR ν_max_: 3247, 1656, 1599, 1519; UV (MeOH) cm^−1; 1^H NMR (500 MHz, CDCl_3_): δ 3.96 (3H, s, C_6_–OCH_3_), 6.60 (1H, d, *J* = 16.0 Hz, H-2), 6.97 (1H, d, *J* = 8.0 Hz, H-8), 7.13 (1H, dd, *J* = 8.0, 2.0 Hz, H-9), 7.16 (1H, d, *J* = 2.0 Hz, H-5), 7.41 (1H, d, *J* = 16.0 Hz, H-3), 9.66 (1H, s, CHO); ESI-MS *m*/*z*: 178 [M]^+^.

Vanillin (**12**). Yellow powder (CH_2_Cl_2_); UV λ_max_: 205, 230, 280, 310 nm; IR ν_max_: 3350, 1595, 1290, 725 cm^−1; 1^H NMR (500 MHz, CDCl_3_): δ 3.97 (3H, *s*, C_3_–OCH_3_), 6.22 (1H, br s, OH), 7.04 (1H, d, *J* = 8.5 Hz, H-5), 7.42 (1H, d, *J* = 2.0 Hz, H-2), 7.44 (1H, dd, *J* = 8.5, 2.0 Hz, H-6), 9.83 (1H, s, CHO); ESI-MS *m*/*z*: 152 [M]^+^.

Vanillic acid (**13**). Colorless needles (acetone), UV λ_max_: 220, 265, 300 nm, IR ν_max_: 3550, 1680, 1510, 1280 cm^−1; 1^H NMR (500 MHz, CDCl_3_): δ 3.97 (3H, s, C_3_–OCH_3_), 6.97 (1H, d, *J* = 8.0 Hz, H-5), 7.58 (1H, d, *J* = 2.0 Hz, H-2), 7.70 (1H, dd, *J* = 8.0, 2.0 Hz, H-6); ESI-MS *m*/*z*: 168 [M]^+^.

Methyl vanillate (**14**). Colorless needles (CH_2_Cl_2_); UV λ_max_: 221, 263, 293 nm; IR ν_max_: 3300, 1716, 1595, 1518 cm^−1; 1^H NMR (500 MHz, CDCl_3_): δ 3.96 (3H, s, C_3_–OCH_3_), 3.99 (3H, s, COOCH_3_), 6.20 (1H, br s, OH), 6.97 (1H, dd, *J* = 8.5, 2.0 Hz, H-6), 7.58 (1H, d, *J* = 2.0 Hz, H-2), 7.70 (1H, d, *J* = 8.5 Hz, H-5); ESI-MS *m*/*z*: 182 [M]^+^.

*p*-Hydroxybenzoic acid (**15**). Brown powder (CH_2_Cl_2_), UV λ_max_: 250, 285, 290 nm, IR ν_max_: 3500, 1590, 1260, 760 cm^−1; 1^H NMR (500 MHz, CDCl_3_): δ 6.88 (2H, d, *J* = 8.5 Hz, H-3 and H-5), 7.99 (2H, d, *J* = 8.5 Hz, H-2 and H-6); ESI-MS *m*/*z*: 138 [M]^+^.

syringic acid (**16**). Brown needles (CH_2_Cl_2_), UV λ_max_: 212, 235, 308 nm, IR ν_max_: 3255, 1670, 1514, 1330 cm^−1; 1^H NMR (500 MHz, CDCl_3_): δ 3.96 (6H, s, C_3_–OCH_3_ and C_5_–OCH_3_), 7.39 (2H, s, H-2 and H-6); ESI-MS *m*/*z*: 198 [M]^+^.

2,6-Dimethoxy-*p*-quinone (**17**). Yellow needles (CH_2_Cl_2_); UV λ_max_: 285 nm; IR ν_max_: 1695, 1595, 1259, 1109 cm^−1; 1^H NMR (500 MHz, CDCl_3_): δ 3.97 (6H, s, C_2_–OCH_3_ and C_6_–OCH_3_), 5.98 (2H, s, H-3 and H-5); ESI-MS *m*/*z*: 168 [M]^+^.

### 3.4. Reagents and Materials

Vitamin C, dimethyl sulfoxide (DMSO), 1,1-diphenyl-2-picrylhydrazyl (DPPH), ethylene diamine tetra-acetic acid (EDTA), 2,6-di-*tert*-butyl-4-methylphenol (BHT), nitro bluetetrazolium (NBT), 2,4,6-tripyridyl-*S*-triazine (TPTZ), phenazine methosulfate (PMS), nicotinamide adenine dinucleotide (NADH), potassium ferricyanide (K_3_Fe(CN)_6_), trichloroacetic acid, horseradish peroxidase (HRPase), FeCl_3_, FeCl_2_·4H_2_O and MTT were purchased from Sigma Chemical (St. Louis, MO, USA). Fetal bovine serum (FBS) was obtained from GIBCO BRL (Gaithersburg, MD, USA). Dulbecco’s modified Eagle’s medium (DMEM) and nutrient mixture F-12 were purchased from Invitrogen (Technologies, Carlsbad, CA, USA). All buffers and other reagents were of the highest purity commercially available.

### 3.5. Determination of DPPH Radical Scavenging Capacity

Most cosmetics and food compounds have free radical scavenging abilities. The antioxidant activity of testing compounds was measured in terms of hydrogen donating or radical scavenging ability using the stable DPPH method as modified by Wang *et al.* [16]. Proper concentrations of the samples were added to 0.2 mL of DPPH (60 μM) solution. When DPPH reacts with an antioxidant compound that donates hydrogen, it is reduced, resulting in a decrease in the absorbance at 520 nm. The absorbance was recorded at 30 min using a UV-visible spectrophotometer. Vitamin C was used as a positive control. The percentages of remaining DPPH were plotted against the sample to obtain the amount of antioxidant required to reduce the initial concentration of DPPH. Scavenging activity (%) was determined as

(1) $$\text{Scavenging activity }(\%)=100\times (OD_{\text{control}}-OD_{\text{sample}})/OD_{\text{control}}$$

### 3.6. ABTS^+^ Cation Radical Scavenging Assay

The scavenging activity of ABTS^+^ was measured according to the method described by Re *et al.* with minor modifications [23]. The ABTS radical stock was stable in this form for more than two days when stored in the dark at room temperature. Briefly, ABTS was dissolved in deionized water to 7 mM in concentration (pH = 7.4) and was then mixed with 2.45 mM potassium persulfate. The scavenging activity was determined by mixing with 180 μL of ABTS and 40 μL of testing samples, and followed by measuring at absorbance 734 nm at 10 min. We applied vitamin C as a positive control and phosphate buffered saline as a negative control, and the calculation formula was similar to Equation 1.

### 3.7. Metal Chelating Activity

The ferrous ion-chelating potential of chlorophyll was investigated according to the method described by Wang *et al.* [16] Briefly, testing samples at suitable concentrations dissolved in DMSO were added to a solution of 2.0 mM FeCl_2_·4H_2_O (0.05 mL). The reaction was initiated by the addition of 5 mM ferrozine (0.2 mL), and the mixture was vigorously shaken and left standing at room temperature for 10 min. After the mixture reached equilibrium, the absorbance of the mixture was measured at 560 nm against a blank. EDTA was used as a positive control, and the chelating activity calculation formula was similar to Equation 1.

### 3.8. Reducing Power

The reducing powers of our natural pure compounds were determined according to the method of [15]. Briefly, various concentrations of test samples were mixed with 67 mM phosphate buffer (pH 6.8, 0.085 mL) and 20% potassium ferricyanide [K_3_Fe(CN)_6_, 2.5 μL) The mixture was incubated at 50 °C for 20 min, and trichloroacetic acid (10%, 0.16 mL) was then added to the mixture that was then centrifuged for 10 min at 3000*g*. The upper layer of the solution (75 μL) was mixed with 2% FeCl_3_ (25 μL), and the absorbance was measured with a 96-well plate spectrophotometer at 700 nm. Butylated hydroxyanisole (BHA) was used as a positive control. A higher absorbance demonstrates a higher reductive capability.

### 3.9. Assay on Mushroom Tyrosinase Activity

Tyrosinase inhibitory activity was determined spectrophotometrically according to the method described previously [22], with minor modifications. Assays were conducted in a 96-well microplate, an ELISA plate reader (Molecular Devices) being used to determine the absorbance at 490 nm. Kojic acid was used as a positive control. The test substance was dissolved in aqueous DMSO, and incubated with L-tyrosine (2.5 mg/mL) in 50 mM phosphate buffer (pH 6.8). Then, 25 U/mL of mushroom tyrosinase in the same buffer was added, and the mixture was incubated at 37 °C for 30 min. Tyrosinase inhibitory activity was determined at 490 nm by the following equation:

(2) % Inhibition=100%×((A-B)-(C-D))/(A-B)

where *A* is the optical density (*OD*_490_) without test substance; *B* is the *OD*_490_ without test substance, but with tyrosinase; *C* is the *OD*_490_ with test substance; and *D* is the *OD*_490_ with test substance, but without tyrosinase. The results are listed in Table 1.

### 3.10. Cell Culture

Melanoma B16 cells (CRL 6323) were obtained from ATCC (Manassas, VA, USA), cultured in DMEM (13.4 mg/mL Dulbecco’s modified Eagle’s medium, 10 mM HEPES, 143 U/mL benzylpenicillin potassium, 100 Lg/mL streptomycin sulfate and 24 mM NaHCO3, pH 7.1) containing 10% FBS, 1% P/S and incubated at 37 °C under 5% CO_2_ atmosphere. The culture procedures were similar to our previous paper with minor modifications [24].

### 3.11. Cell Viability

Cell viability was determined using the MTT assay [22]. The method is based on the ability of a mitochondrial dehydrogenase from viable cells to cleave the tetrazolium rings of the pale yellow MTT and form impermeable crystals of a dark-blue formazan, thus resulting in accumulation within healthy cells. Briefly, cells were seeded in 96-well plates at a density of 8 × 10^3^ cells/well. The medium was then changed, and cells were maintained in either solvent alone (control cells) or in the presence of the indicated folic acid derivative in a final volume of 100 μL in 10% FBS culture medium. Each sample was added to a micro-plate and incubated under the same conditions as above for 24 h. After 24 h of incubation, the medium was replaced with 100 μL of fresh medium, including 0.5 mg/mL MTT. The plate was cultured in a 37 °C incubator filled with 5% CO_2_ for 2 h. Each precipitate in a specific dish was dissolved in 100 μL of DMSO to dissolve the purple formazan crystals. After the dishes were gently shaken for 10 min in the dark to ensure maximal dissolution of formazan crystals, the absorbance (*A*) values of the supernatant were measured at 595 nm. Cell growth was calculated as

(3) $$\text{Cell growth}=(A_{\text{sample}}-A_{\text{blank}})/(A_{\text{control}}-A_{\text{blank}})\times 100\%$$

### 3.12. Melanin Quantification

Briefly, we followed the previous method with minor modifications [21,22]. Melanoma B16 cells were seeded at a density of 2.5 × 10^5^ cells/well of 6-well culture plates in 1500 μL of medium containing various concentrations of testing samples and incubated at 37 °C under 5% CO_2_ atmosphere for 48 h. Cell pellets were dissolved in 2.0 N NaOH containing 10% DMSO and heated at 80 °C for 1 h, and suspensions were clarified by centrifugation for 10 min at 10,000*g*. Amounts of melanin in the NaOH solution were spectrophotometrically measured at 405 nm. The control group had no additional agents.

### 3.13. Tyrosinase Assay

The tyrosinase activity was estimated by measuring the rate of dopachrome formation, based on the method described previously with minor modifications [21]. B16F10 cell were placed in 24-well plates in 500 μL of medium containing various concentrations of testing samples and incubated for 2 days. The sample-treated cells were washed with phosphate-buffered saline (PBS) and lysed with 1% Triton. The enzyme extract of cellular lysate was added to 10 μL of 10 mm L-tyrosine and 10 mm L-dopa as substrates mixed in 0.1 m phosphate buffer (pH 6.8). This reaction was then incubated at 37 °C for 3 h in a dark environment, and the absorbance at 490 nm was measured on a spectrophotometer. The absorbance after 3 h incubation was in direct proportion to the dopachrome formation rate [22], and the control group had no additional agents.

### 3.14. Statistical Analysis

All determinations were carried out at least three times, and in triplicate, on each occasion and at each separate concentration of the standard and samples. The results were expressed as the average of the mean values ± standard deviation (SD), and statistical comparisons were carried out using the Student’s *t*-test.

## 4. Conclusions

This study reported the antioxidant, mushroom tyrosinase inhibition, tyrosinase activity and melanin content in B16F10 cells of **17** pure constituents from *L. tulipifera*. The results revealed that some of the seventeen compounds showed potential antioxidant and skin whitening abilities. Within anti-oxidative assessments: compound **2** showed the highest antioxidant power of seventeen compounds on DPPH free radical scavenging assay; compounds **1**–**3**, **6**, **9**, **10**, **12**, **13**, **16** and **17** exhibited antioxidant competences on ABTS free radical scavenging test. On metal chelating power examination, compounds **3**, **8**, **10** and **17** were moderately effective, and on reducing power, compounds **2** and **9** showed middle to high abilities. Within tyrosinase inhibitory assessments, we discovered the non-cytotoxicity properties of seventeen compounds in B16F10 cells, hence, we continued the whitening abilities. And, we found compounds **2**–**4**, **12** and **13** reduced tyrosinase activities and melanin contents, which means the compounds had potential for de-pigmentation. Importantly, compound **2** and **3** showed not only antioxidant but also de-pigmentation abilities without apparent cytotoxicity in our experiment. Today, more and more people pay attention to multi-functional cosmetic ingredients. For this reason, compound **2** and **3** were great potential candidates for the cosmetic business, therapeutic applications and the food industry.

## Figures and Tables

**Table 1:** Antioxidative properties of compounds isolated from *Liriodendron tulipifera* determined by various assay. All compounds, including controls, were used at 100 μM. (-), no testing; (ns), no significance.

Compounds	DPPH (%)	ABTS (%)	Chelating (%)	Reducing power (OD700)
Vitamin C a	88.6 ± 1.8	76.4 ± 5.6	-	-
EDTA b	-	-	86.9 ± 4.5	-
BHA c	-	-	-	0.98 ± 0.1
(−)-Eudesmin (**1**)	ns	64.1 ± 5.6	ns	0.28 ± 0.0
(+)-Syringaresinol (**2**)	38.5 ± 4.8	84.8 ± 7.3	ns	0.66 ± 0.3
(+)-Yangambin (**3**)	ns	28.1 ± 6.7	13.5 ± 0.6	0.24 ± 0.0
β-Sitosterol (**4**)	ns	ns	ns	ns
Stigmasterol (**5**)	ns	ns	ns	ns
β-Sitostenone (**6**)	ns	52.4 ± 7.4	ns	0.29 ± 0.0
Stigmastenone (**7**)	ns	ns	ns	ns
Methyl 4-hydroxy-2-methylbenzoate (**8**)	ns	ns	17.4 ± 9.1	0.18 ± 0.0
β-Orcinol carboxylate (**9**)	ns	79.8 ± 2.2	ns	0.45 ± 0.0
Methyl haematommate (**10**)	ns	72.5 ± 6.1	19.8 ± 8.3	0.28 ± 0.0
Coniferyl aldehyde (**11**)	ns	ns	ns	0.24 ± 0.0
Vanillin (**12**)	ns	19.4 ± 5.5	ns	0.24 ± 0.0
Vanillic acid (**13**)	ns	22.7 ± 5.2	ns	0.18 ± 0.0
Methyl vanillate (**14**)	ns	ns	ns	ns
*p*-Hydroxybenzoic acid (**15**)	ns	ns	ns	ns
Syringic acid (**16**)	ns	49.4 ± 10.8	ns	0.25 ± 0.0
2,6-Dimethoxy-*p*-quinone (**17**)	ns	31.5 ± 13.5	25.5 ± 6.8	0.29 ± 0.0

Data were expressed as a mean value of at least three independent experiments.

a Vitamin C was used as a positive control on DPPH assay at 100 μM;

b EDTA was used as a positive control on metal chelating ability at 100 μM;

c BHA was used as a positive control on reducing power at 100 μM.

**Table 2:** Inhibitions of *L. tulipifera* compounds on mushroom tyrosinase at 100 μM. (ns), no significance.

*L. tulipifera* compounds	Mushroom tyrosinase inhibition (%)
Kojic acid a	89.2 ± 0.1
(−)-Eudesmin (**1**)	ns
(+)-Syringaresinol (**2**)	ns
(+)-Yangambin (**3**)	ns
β-Sitosterol (**4**)	ns
Stigmasterol (**5**)	ns
β-Sitostenone (**6**)	ns
Stigmastenone (**7**)	ns
Methyl 4-hydroxy-2-methylbenzoate (**8**)	23.2 ± 0.4
β-Orcinol carboxylate (**9**)	ns
Methyl haematommate (**10**)	ns
Coniferyl aldehyde (**11**)	ns
Vanillin (**12**)	ns
Vanillic acid (**13**)	22.8 ± 0.4
Methyl vanillate (**14**)	ns
*p*-Hydroxybenzoic acid (**15**)	15.4 ± 0.4
Syringic acid (**16**)	10.6 ± 0.4
2,6-Dimethoxy-*p*-quinone (**17**)	ns

Data were expressed as a mean value of at least three independent experiments.

a Kojic acid was used as a positive control of mushroom tyrosinase assay at 100 μM.

## References

[1] Kim T.W. (1995). The Woody Plants of Korea in Color.

[2] Merkle S.A., Sommer H.E., Bajaj Y.P.S. (1991). Yellow-Poplar (*Liriodendron* spp.). Biotechnology in Agriculture and Forestry, Trees III.

[3] Muhammad I., Hufford C.D. (1989). Phenylpropanoids, sesqiterpenes, and alkaloids from the seeds of *Liriodendron tulipifera*. J. Nat. Prod.

[4] Xu Z.H., Qin G.W., Li X.Y., Xu R.S. (2011). New biflavanones and bioactive compounds from *Stellera chamaejasme* L. Yao Xue Xue Bao.

[5] Kelm M.A., Nair M.G. (2000). A brief summary of biologically active compounds from *Magnolia* spp. Nat. Prod. Chem.

[6] Chen C.Y., Cheng M.J., Chiang Y.J., Bai J.C., Chiu C.T., Lin R.J., Hsui Y.R., Lo W.L. (2009). Chemical constituents from the leaves of *Machilus zuihoensis* Hayata var. *mushaensis* (Lu) Y.C. Liu. Nat. Prod. Res.

[7] Baldé A.M., Apers S., de Bruyne T.E., van den Heuvel H., Claeys M., Vlietinck A.J., Pieters L.A. (2000). Steroids from *Harrisonia abyssinica*. Planta Med.

[8] Piao Y.Z., Kim Y.J., Kim Y.A., Lee H.S., Hammock B.D., Lee Y.T. (2009). Development of ELISAs for the class-specific determination of organophosphorus pesticides. J. Agric. Food Chem.

[9] Rojas I.S., Lotina-Hennsen B., Mata R. (2000). Effect of lichen metabolites on thylakoid electron transport and photophosphorylation in isolated spinach chloroplasts. J. Nat. Prod.

[10] Shi H., Wang H., Wang M., Li X. (2009). Antioxidant activity and chemical composition of *Torreya grandis* cv. Merrillii seed. Nat. Prod. Commun.

[11] Chen C.Y., Chang F.R., Wu Y.C. (1999). Cheritamine, a new N-Fatty acyl tryamine and other constituents from the stems of *Annona cherimola*. J. Chin. Chem. Soc.

[12] Wang H.M., Chou Y.T., Hong Z.L., Chen H.A., Chang Y.C., Yang W.L., Chang H.C., Mai C.T., Chen C.Y. (2011). Bioconstituents from stems of *Synsepalum dulcificum* Daniell (Sapotaceae) inhibit human melanoma proliferation, reduce mushroom tyrosinase activity and have antioxidant properties. J. Taiwan Inst. Chem. Eng.

[13] Chen C.Y., Cheng K.C., Chang A.Y., Lin Y.T., Hseu Y.C., Wang H.M. (2012). 10-Shogaol, an antioxidant from *Zingiber officinale* for skin cell proliferation and migration enhancer. Int. J. Mol. Sci.

[14] Wang H.M., Cheng K.C., Lin C.J., Hsu S.W., Fang W.C., Hsu T.F., Chiu C.C., Chang H.W., Hsu C.H., Lee A.Y. (2010). Obtusilactone A and (−)-sesamin induce apoptosis in human lung cancer cells via inhibiting mitochondrial Lon protease and activating DNA damage checkpoints. Cancer Sci.

[15] Chiu C.C., Chou H.I., Wu P.F., Wang H.M., Chen C.Y. (2012). Bio-functional constituents from the stems of *Liriodendron tulipifera*. Molecules.

[16] Wang H.M., Pan J.L., Chiu C.C., Chen C.Y., Yang M.H., Chang J.S. (2010). Identification of anti-lung cancer extract from *Chlorella vulgaris* C–C by antioxidant property using supercritical carbon dioxide extraction. Process Biochem.

[17] Liao W.T., Huang T.S., Chiu C.C., Pan J.L., Liang S.S., Chen B.H., Chen S.H., Liu P.L., Wang H.C., Wang H.M. (2012). Biological properties of acidic cosmetic water from seawater. Int. J. Mol. Sci.

[18] Chen C.Y., Kuo P.L., Chen Y.H., Huang J.C., Ho M.L., Lin R.J., Chang J.S., Wang H.M. (2010). Tyrosinase inhibition, free radical scavenging, antimicrobial and anticancer proliferation activities of *Sapindus mukorossi* extracts. J. Taiwan Inst. Chem. Eng.

[19] Wang H.M., Chen C.Y., Chen H.A., Huang W.C., Lin W.R., Chen T.C., Lin C.Y., Chien H.J., Lu P.L., Lin C.M. (2010). *Zingiber officinale* (ginger) compounds with tetracycline have synergistic effects against clinical extensively-drug resistant *Acinetobacter baumannii*. Phytother. Res.

[20] Chen B.H., Chang H.W., Huang H.M., Chong I.W., Chen J.S., Chen C.Y., Wang H.M. (2011). (−)-Anonaine induces oxidative stress and DNA damage to inhibit growth and migration of human lung carcinoma H1299 cells. J. Agric. Food Chem..

[21] Wang H.M., Chen C.Y., Chen C.Y., Ho M.L., Chou Y.T., Chang H.C., Lee C.H., Wang C.Z., Chu I.M. (2010). (−)-*N*-Formylanonaine from *Michelia alba* as a human tyrosinase inhibitor and antioxidant. Bioorg. Med. Chem..

[22] Wang H.M., Chen C.Y., Wen Z.H. (2011). Identifying melanogenesis inhibitors from *Cinnamomum subavenium* with *in vitro* and *in vivo* screening systems by targeting the human tyrosinase. Exp. Dermatol.

[23] Re R., Pellegrini N., Proteggente A., Pannala A., Yang M., Rice-evans C. (1999). Antioxidant activity applying an improved abts radical cation decolorization assay. Free Radical Biol. Med.

[24] Wang H.M., Chiu C.C., Wu P.F., Chen C.Y. (2011). Subamolide E from *Cinnamomum subavenium* induces sub G1 cell cycle arrest, caspase-dependent apoptosis, and reduces migration ability of human melanoma cells. J. Agric. Food Chem.

